# Supplementary material for: Patterns of fear of crime: A mixed-methods exploration of the model of experiential and expressive fear of crime
Source: PLoS One. 2025 Sep 2;20(9):e0329493. doi: 10.1371/journal.pone.0329493 (PMC12404561; doi:10.1371/journal.pone.0329493)
Supplement: S1 Table — The first column presents the three main categories of variables, the second lists the questions and their response modalities, and the third specifies the assctive variables included in the multivariate configuration analyses (Multiple Correspondence Analysis and Cluster Analysis). (DOCX) [file pone.0329493.s001.docx]

**S1 Table: The variables integrated into the analysis as active variables.**

| **Categories** | **Questions and modalities** | **Variable** |
| --- | --- | --- |
| Social and political attitudes and values | Priority problem in French society | Health |
|  |  | Environment |
|  |  | Terrorism |
|  |  | Unemployment |
|  |  | Crime |
|  |  | Poverty |
|  |  | Road safety |
|  |  | Racism, discrimination |
|  | Authoritarian attitudes toward law and order (1 = Strongly agree; 4 = Strongly disagree) | Young people today don't have enough respect for traditional French values |
|  |  | People who break the law should be given stiffer sentences |
|  |  | Schools should teach children to obey authority |
|  | The main cause of crime | Poverty |
|  |  | Unemployment |
|  |  | Lack of family support |
|  |  | Lack of school support |
|  |  | Lack of justice sanction |
|  |  | Shortfall in prison capacity |
|  |  | Informal economy |
|  |  | Too much media coverage |
|  | If we could spend more money to fight crime, should we, in priority? | Build new prisons |
|  |  | Help and compensate victims more |
|  |  | Develop youth prevention |
|  |  | Recruit more police officers |
| Perceptions of the local environment | Attitudes toward social change in the community (1 = Had increased; 2 = Not changed; 3 = Had decreased) | A sense of belonging to the community |
|  |  | A sense of shared values amongst people who live here |
|  |  | A sense of right and wrong amongst people who live here |
|  |  | Young people's respect for rules and authority |
|  |  | Young people's respect for other people and their quality of life |
|  | Perception of problems in neighborhood (0 = not a problem; 1 = problem) | Noise |
|  |  | Pollution |
|  |  | Lack of public transportation |
|  |  | Lack of infrastructure |
|  |  | Lack of entertainment |
|  |  | Lack of cleanliness |
|  |  | Crime |
|  |  | Lack of road safety |
|  |  | Bad reputation |
|  |  | Drug |
|  |  | Youth gangs |
|  |  | Domestic violence |
|  | The concern about social cohesion (0 = no; 1 = yes) | This area has a close, tight-knit community |
|  |  | This area is a friendly place to live |
|  |  | This area is a place where local people look after each other |
|  |  | I am proud to live in this neighborhood |
|  |  | People in this area are trustworthy |
|  | The concern about collective efficacy (0 = no; 1 = yes). I can rely on my neighbors... | To make a purchase |
|  |  | To babysit a child for a few hours |
|  |  | To call the emergency services if needed |
|  |  | To help me find a job |
|  |  | To help me get around |
|  |  | To notify the police if necessary |
| Perceived likelihood, victimization and worry about crime | Perceived likelihood of being victimized (1 = very unlikely; 4 = very likely) | Vehicle theft |
|  |  | Larceny |
|  |  | Sexual assault |
|  |  | Physical assault |
|  |  | Verbal assault |
|  |  | Burglary |
|  | Worry about crime in the past year (1 = Never; 2 = Once; 3 = More than one time) | Vehicle theft |
|  |  | Larceny |
|  |  | Sexual assault |
|  |  | Physical assault |
|  |  | Verbal assault |
|  |  | Burglary |
|  | Direct victimization in the past year (1= Never; 2 = One; 3 = More than one time) | Bike theft |
|  |  | Motorbike theft |
|  |  | Physical assault |
|  |  | Sexual assault |
|  |  | Threats |
|  |  | Insults |
|  |  | Burglary |
|  |  | House vandalism |
|  |  | Vehicle vandalism |
|  |  | Car theft |

The first column presents the three main categories of variables, the second lists the questions and their response modalities, and the third specifies the active variables included in the multivariate configuration analyses (Multiple Correspondence Analysis and Cluster Analysis).
