# Supplementary material for: Patterns of fear of crime: A mixed-methods exploration of the model of experiential and expressive fear of crime
Source: PLoS One. 2025 Sep 2;20(9):e0329493. doi: 10.1371/journal.pone.0329493 (PMC12404561; doi:10.1371/journal.pone.0329493)
Supplement: Attachment — Submitted filename: Response to Reviewers.docx [file pone.0329493.s004.docx]

Response to Reviewers

1. Structural and Organizational Concerns:
- The authors are encouraged to amend the fragmented and confusing structure of the paper, they are recommended to completely reorganize the manuscript into clearly defined sections (e.g., Introduction, Theoretical Framework, Study Design, Results, Discussion, Conclusion).

The paper has been completely reorganized following the recommendations of the first reviewer (see the responses to the first reviewer's comments).

- The excessive use of subheadings creates unnecessary complexity and redundancy, which detracts from the paper’s readability and coherence. Simplifying and synthesizing sections (e.g., merging theoretical framework and earlier versions of the model) is critical.

The requests for the removal of subtitles have all been followed. In accordance with the recommendations of the first reviewer, we have replaced the subtitles with bullet points. More generally, all proposed structural modifications have been adopted (see the responses to the first reviewer’s comments).

However, regarding the request to summarize the sections, we emphasize that the method used in this article is not deployed elsewhere. It is therefore crucial to explain it thoroughly. This article should be seen as a foundation for further analyses. With this objective in mind, PLOS One appeared to be a particularly suitable choice. Indeed, your journal offers the significant advantage of allowing researchers to provide detailed explanations of their methodologies and results without imposing any word limit. That being said, we have made every effort to be as concise as possible.

2. Methodological Clarity and Rigor:
- The methodology is unclear and requires restructuring into distinct subsections (Sample, Measures, Models, and Data Analysis). Additionally, specific elements like the logistic model equation and a consistent taxonomic criterion for group categorizations need to be clearly explained and justified.

The methodology section has been restructured according to the first reviewer's recommendations. The regression model equation was already present in the first version of the article. However, we have modified the way the equation's terms are presented to improve readability.

Regarding the classification analysis, we have added a section to explain why no taxonomic criterion is defined a priori. We have thus provided a clearer explanation of the inductive approach of the method used (see the responses to the first reviewer's comments).

- Authors should provide sufficient detail on effect sizes and the significance of sociodemographic predictors, which undermines the credibility of the results.

The "Limitations" section has been revised and expanded to provide more detail (see the responses to the first and second reviewers' comments).

3. Presentation of Results:
- The presentation of results is dense and difficult to digest. Tables and figures are either unreadable or redundant (e.g., Table 6 with low R2R^2 and Figure 1). Authors are advised to consolidate results into fewer, more meaningful tables and moving supplementary details to an appendix is necessary.

Since all the presented results are statistically significant, it is difficult to select which ones should remain in the main text and which should be placed in the appendix. One of the solutions suggested by the first reviewer was to subdivide the densest tables into multiple parts. We have therefore adopted this approach (see the responses to the first reviewer’s comments).

- Authors are also recommended to synthesize key findings in a simple table within the discussion for better clarity.

Table 8 has been created to address this request. It summarizes our main results while comparing them to those of previous versions of the EEF model (see the responses to the first reviewer’s comments).

4. Discussion and Implications:
- There is a need for a more critical synthesis and deeper analysis in the discussion. A concise explanation of results, highlighting unexpected or profound findings, and their relevance to existing literature.

The "Discussion" section has been extensively revised to address this comment. In addition to the creation of Table 8, the section has been reorganized, and bullet points have been added to highlight the key contributions of our results compared to previous studies. The hypotheses—integrated into the "Theoretical Framework" section following the first reviewer’s advice—have been discussed in light of the obtained results. Finally, this section now includes the references suggested by the first reviewer (see the responses to the first and second reviewers’ comments).

- Practical and policy implications are underdeveloped, insufficient elaboration on public policy implications and generalizability of findings to other contexts.

Following the recommendations of both reviewers, we have expanded the section on policy implications (see the responses to the first and second reviewers’ comments). Additionally, in the conclusion (section "Ideas for Future Research"), we discuss the possibility of generalizing our findings to other geographical and cultural contexts (see the responses to the second reviewer’s comments).

5. Theoretical Framework:
- Authors are also encouraged to improve the theoretical framework by incorporating relevant suggested readings to provide a stronger foundation for the analysis. Additionally, previous versions of the model should be clearly synthesized into a cohesive narrative.

The references suggested by the first reviewer have been included in the "Discussion" section rather than in the "Theoretical Framework" section. Given their content, we considered that they were more appropriately placed in the discussion.

Additionally, following the first reviewer’s advice, we have created a table (Table 1) that summarizes the key results from previous versions of the EEF model.

- The theoretical contribution of the paper is unclear without these improvements.

Indeed, all these modifications significantly improve the clarity and readability of the paper (see the responses to the first reviewer’s comments).

6. Limitations and Conclusions:
- Authors are advised to place limitations in the conclusion and focus on theoretical, policy, and future research implications. Subsections within the conclusion can improve clarity and emphasize the paper’s contribution to the field.

If the editor and reviewers have no objections, we would prefer to leave the "Limitations" section separate from the "Conclusion." This solution offers two advantages. First, it prevents the conclusion from becoming overly lengthy (which now contains three sub-sections in bullet-point format). Second, it highlights the limitations of a method that is not used elsewhere. The "Limitations" section also aims to reflect on ways to address these issues in future studies (see the responses to the first reviewer’s comments).

7. Scope and Generalizability:
- One of the main concerns is the lack of discussion on the generalizability of findings beyond the French context. Explicitly addressing how the findings may or may not apply to other cultural or geographical contexts will enhance the paper's broader relevance.

This point is addressed at the very end of the article.

***Comments from PLOS Editorial Office:*** *We note that one or more reviewers has recommended that you cite specific previously published works. As always, we recommend that you please review and evaluate the requested works to determine whether they are relevant and should be cited. It is not a requirement to cite these works. We appreciate your attention to this request.*


[Note: HTML markup is below. Please do not edit.]

Reviewers' comments:

Reviewer's Responses to Questions

**Comments to the Author**

1. Is the manuscript technically sound, and do the data support the conclusions?

The manuscript must describe a technically sound piece of scientific research with data that supports the conclusions. Experiments must have been conducted rigorously, with appropriate controls, replication, and sample sizes. The conclusions must be drawn appropriately based on the data presented.

Reviewer #1: Partly

Reviewer #2: Yes

2. Has the statistical analysis been performed appropriately and rigorously?

Reviewer #1: Yes

Reviewer #2: Yes

3. Have the authors made all data underlying the findings in their manuscript fully available?

The [PLOS Data policy](http://track.editorialmanager.com/CL0/http:%2F%2Fwww.plosone.org%2Fstatic%2Fpolicies.action%23sharing/1/010f01946af6afc3-35e99ea9-96d7-4b94-a738-eb6045bd96ac-000000/Tg7FYn1goIr5iOmrG06dTYxDMdQFWBDxeRrrHq6V9yU=193) requires authors to make all data underlying the findings described in their manuscript fully available without restriction, with rare exception (please refer to the Data Availability Statement in the manuscript PDF file). The data should be provided as part of the manuscript or its supporting information, or deposited to a public repository. For example, in addition to summary statistics, the data points behind means, medians and variance measures should be available. If there are restrictions on publicly sharing data—e.g. participant privacy or use of data from a third party—those must be specified.

Reviewer #1: No

Reviewer #2: Yes

4. Is the manuscript presented in an intelligible fashion and written in standard English?

PLOS ONE does not copyedit accepted manuscripts, so the language in submitted articles must be clear, correct, and unambiguous. Any typographical or grammatical errors should be corrected at revision, so please note any specific errors here.

Reviewer #1: Yes

Reviewer #2: Yes

5. Review Comments to the Author

Please use the space provided to explain your answers to the questions above. You may also include additional comments for the author, including concerns about dual publication, research ethics, or publication ethics. (Please upload your review as an attachment if it exceeds 20,000 characters)

**Reviewer #1: Patterns of fear of crime. Revisit and synthesize the model of experiential and expressive fear of crime**

The topics of this paper are interesting, though well known. The structure and content must be revised, and results have to be better explained by authors before to be reconsidered.

Title has to be shorter, clarify if this is a case study in France and the period.

We have modified the title of the article. Instead of " Patterns of fear of crime. Revisit and synthesize the model of experiential and expressive fear of crime," we propose " Patterns of fear of crime: A mixed-methods exploration of the model of experiential and expressive fear of crime." This title emphasizes the originality of the paper, which is based on two statistical analysis methods, the combination of which is rarely used in the study of fear of crime.

Regarding the inclusion of the country in which the study was conducted, we would prefer not to mention it in the title. Aside from the fact that this information would further lengthen the title, we believe it could reduce the interest in the article by suggesting, from the title, that the results would only apply to the case of France. As the paper’s focus is on identifying the structures of relationships between variables (rather than, for example, presenting prevalences), we believe the results can extend beyond the French context.

Abstract has to be shorter, clarify the goal, what the study adds and implications for criminal justice system. Also here clarify if data are related to France and period under study.

We have revised the abstract following the reviewer’s suggestions. The contributions of the study to 1) research and 2) the criminal justice system have been reformulated, while adhering to the word limit imposed by the journal (300 words).

The paper is long and has to be reduced. The structure is too fragmentated and confusing.

The reviewer details these points in the following comments. We will address our modifications as we go through their remarks.

Authors have to structure the paper as follows.
-Introduction
-Theoretical framework
-Proposed model and Study design
-Results
-Discussion
-Conclusion
Authors have to avoid subheadings that create fragmentation and confusion. If necessary, can use bullet points (same comments for all sections).

We have adopted the structure suggested by the reviewer. We have removed the subtitles and replaced them with bullet points.

Introduction has to better clarify the research questions of this study and provide more theoretical background about these topics, the socioeconomic drivers of crime in society, etc. After that they can focus on the topics of this study to provide a correct analysis for fruitful discussion. (See suggested readings that must be all read and used in the text).

As requested, the research question has been explicitly stated. We have also revised the wording of the article's objectives. Two bullet points have been added to highlight these objectives.

However, to avoid overloading the text and to prevent repetition with the subsequent sections, we prefer to incorporate the suggested works into the main body of the text at appropriate points, rather than strengthening the theoretical background in the introduction. Indeed, the theoretical background for the EEF model is quite extensive. As mentioned in the introduction, this unifying model "integrates, from an updated literature review, the 'factors' recognized by previous studies as being the most contributive to this social phenomenon." These factors are numerous, and we intentionally limit the introduction to this description to avoid repetition with the next section (theoretical framework), which goes into detail about the origins of the model.

Sections From the model's origins to its design, The results of the first version of the model, The results of the second version of the model, The results of the third version of the model can be merged and called “theoretical framework” reducing the content. A figure can synthetize these results of the previous version of the model to be clear for readers.

The "Theoretical Framework" section has been created. The sections "From the model's origins to its design," "The results of the first version of the model," "The results of the second version of the model," and "The results of the third version of the model" have been integrated into the "Theoretical Framework" section and are now presented in bullet points.

Table 1, titled "Comparative Synthesis of the Three Versions of the EEF Model," has been added at the end of the section. As requested, it summarizes the main methodological characteristics and key results of the three versions of the EEF model.

Section Hypothetico-Deductive Reasoning to Inductive Reasoning can be included in the study design. Reduce the section and provide a clear working hypothesis alternative or complementary to previous approaches.

The content of the section "Hypothetico-Deductive Reasoning to Inductive Reasoning" has been integrated at the beginning of the "Proposed Model and Study Design" section. We have placed a footnote (85) regarding the difficulty for an SEM model to identify multiple relationships between variables. This helps significantly reduce the content of the section.

Additionally, we have explicitly written our three working hypotheses, which are placed at the end of the section in bullet points. Finally, to reduce the confusion mentioned by the reviewer, the subtitle "Hypothetico-Deductive Reasoning to Inductive Reasoning" has been removed.

The methods of this study is not clear. The section of Materials and methods must be re-structured with the following three sections and same order:
• Sample and data
• Measures of variables
• Models and Data analysis procedure. Logistic model has to be specified with equation.
Authors, again, have to avoid subheadings that create fragmentation and confusion. If necessary, you can use bullet points (same comments for section of results and all sections). A flow chart can show the logic of reasoning, indicating expected results (+ or -) to support then with empirical evidence and methods of inquiry to be clear for readers.
Categorization of groups (unworried, ‘worried-dysfunctional, anxious, etc.… hast to be clarified with a consistent taxonomic criterion underpinned in psychological literature.
Be synthetic, details in Appendix.

We have adopted the structure proposed by the reviewer. The "Materials and Methods" section has been renamed "Proposed Model and Study Design." Within this section, the "ELIPSS Panel" section has been renamed "Sample and Data"; the "SPIP2022 Survey" section has been renamed "Measures of Variables"; and the "Data Analysis" section has been renamed "Models and Data Analysis Procedure."

Additionally, the first version of the article already included the regression model equation. However, to improve the understanding of this equation, we have reorganized the presentation of its terms.

Regarding the suggestion to create a flowchart illustrating the logic of the reasoning, we believe it contradicts the inductive reasoning approach we use in this study. Unlike deductive reasoning (which requires making a priori hypotheses about the relationships between variables, thus projecting predefined structures onto the data), inductive reasoning is characterized by the absence of specific hypotheses about the relationships between variables. Therefore, the only hypotheses we formulate are those presented at the beginning of the section. This does not mean that inductive reasoning is without hypotheses (they are necessary to justify the choice of analysis methods), but these hypotheses are not as precise as in deductive reasoning (e.g., the expected effect of one variable on another (+/-)). Furthermore, even if we wanted to, it would be very difficult to detail these expected results, since we aim to highlight several structures of relationships between variables (which means that in two distinct structures, two variables could have very different relationships).

Finally, regarding the categorization of the clusters, the reviewer's comment is very brief, so we are unsure if we fully address the request for clarification. One thing is certain: the grouping procedure used by the classification analysis is inductive (individuals with similar responses are gradually grouped into the same cluster) and not deductive (no a priori taxonomic criteria are defined for grouping individuals into predefined classes). These criteria are subject to post hoc interpretation (which we present and discuss in Figure 2 in the "Discussion" section). We have added a few sentences in the "Data Analysis" section to clarify this point and emphasize the inductive logic of our approach.

Results.
Figure 1 as done cannot be read and it is useless…it can be removed…
To reiterate, avoiding a lot of sub-headings that create fragmentation of the paper.
The paper has a lot of tables that are difficult to digest, some of them can be put in appendix and inserting in the text the most important ones to improve the readability…
Table 1-2-3-5 are long and can be broken in two, one per page, using as title, table 1, table 1 continue, etc.…
Table 6, R2 is low and this can be due to a mis specified model and likely consequential weak and misleading results. Significance can be indicated with stars, as in econometrics textbook, such as ***=0.001, **=0.05, etc.…
Paper dense of information that create confusion.

We believe it is very important to keep the dendrogram (Figure 1), which graphically represents the results of the classification analysis. Indeed, as mentioned previously, the dendrogram reveals the inductive nature of the hierarchical classification analysis. However, we acknowledge that reading this figure can be challenging. Therefore, we have developed a brief description of the dendrogram to help improve its readability and understanding.

Regarding the structure of the text, we have followed the reviewer's recommendations. We have only structured the "Results" section into two subsections. The first is titled "Results of the Classification Analysis," and the second, "Results of the Regression Analysis." Additionally, bullet points have been added to present each of the four classes.

Regarding the tables, the reviewer initially suggested moving some to the annex before later proposing splitting them for easier reading. Since all the results presented are statistically significant, it is difficult to select which ones should remain in the main body of the text and which should be placed in the annex. We propose the solution of separating the tables, while fragmenting them even further. Instead of splitting each table into two parts, we suggest dividing them into three parts. The first part covers the results on perceived probability / crime worry / victimization (e.g., Table 1); the second part covers the perception of the local environment (e.g., Table 1 continued); and the third part focuses on attitudes and social and political values (e.g., Table 1 final). This way, it is possible to place one table per page and position the tables directly next to the relevant text (see the manuscript).

Finally, regarding the table presenting the results of the regression analysis, we have indicated the p-values using the econometric system, as suggested by the reviewer.

Discussion has to be short.

First, authors have to synthesize the main results in a simple table to be clear for readers and then show what this study adds compared to other studies.

See below

Analysis of Findings. Although the Results section provides a detailed description of the data collected, there needs to be a more critical synthesis and comparison of the findings in the discussion.

See below

• Explanation of results: Comment on whether the results were expected for each set of findings; go to greater depth to explain unexpected or incredibly profound findings. If appropriate, note any unusual or unanticipated patterns or trends that emerged from your results and explain their meaning concerning the research problem.

The "Discussion" section has been revised according to the reviewer's suggestions. Figure 2, which graphically presents our results, is now presented at the beginning of the section. The description of this figure has been entirely rewritten to highlight the key findings of the study.

To improve the text's clarity, we sequentially revisit our three hypotheses and demonstrate how our results validate them one by one (this required a reorganization of the text, particularly regarding the development on anxious individuals and the functional worriers. The initial version of the text included a back-and-forth that was difficult to read, which has now been removed).

To clearly emphasize the contributions of our study, the differences with previous versions of the EEF model are presented in bullet points. We have also added a summary table (tab 8) that outlines the main contributions of our model compared to the three previous versions. Finally, several references suggested by the reviewer have been incorporated into this section.

Limitations can be put in conclusion.

If the editor and reviewers have no objections, we would prefer to leave the "Limitations" section separate from the "Conclusion." This solution offers two advantages. First, it prevents the conclusion from becoming overly lengthy (which now contains three sub-sections in bullet-point format). Second, it highlights the limitations of a method that is not used elsewhere. The "Limitations" section also aims to reflect on ways to address these issues in future studies (see the responses to the first reviewer’s comments).

Additionally, we have revised the argument regarding the results (R2) of the regression analysis. We emphasize that our goal is not to obtain the best possible model, but rather to identify the sociodemographic factors of the four classes. We also note that previous versions of the EEF model still obtain even lower scores in this regard. Finally, we propose a suggestion for improving the explanatory power of the sociodemographic variables.

Conclusion has not to be a summary, but authors have to focus on manifold limitations of this study and provide implications to support best practices for criminal justice system.
Make sure you create subsections in the Conclusion: 1) Theoretical Implications, 2) Policy Implications, and 3) Ideas for Future Research.

The conclusion has been restructured according to the reviewer's recommendations. It is now composed of three subsections presented in bullet points: Theoretical Implications, Policy Implications, and Ideas for Future Research. The "Policy Implications" section has been further developed, as has the "Ideas for Future Research" section.

Overall, then, the paper is interesting, but the structure is long and is confused. Theoretical framework can be improved, and some results create confusion… structure of the paper has to be improved; study design, discussion and presentation of results have to be clarified using suggested comments.

I strongly suggest improving the paper, by using all the comments (suggested papers included to read and use all) that I will verify in depth, and maybe it can be considered.
Sure enough , if the paper is not improved as suggested it will be dismissed.

Suggested readings of relevant papers that have to be read and all inserted into the text and references.

Barrantes-Chaves, K. 2024. More than walls: Fear of crime in neighbourhoods with different poverty levels bordering gated communities. The Greater Metropolitan Area of Costa Rica, Cities,154, 105331

This paper has been cited in the conclusion, in the "Theoretical Implications" section. It is mentioned when we discuss the link between poverty and fear of crime. Ref: 156

Syropoulos, S., Leidner, B., Mercado, E., ... Chekroun, P., Rottman, J. 2024. How safe are we? Introducing the multidimensional model of perceived personal safety Personality and Individual Differences, 224, 112640

As this paper links fear of crime and well-being, we have cited it in the "Results" section, specifically in the "Results of the Regression Analysis" section, to discuss the quality of life and distress among the dysfunctional worriers. Ref: 107

Coccia M. 2019. The politics of fear and relationship with the effective level of crime and socioeconomic issues: Empirical analysis of a case study, Journal of Economics Bibliography, Vol 6, No. 4, pp. 357-374, http://dx.doi.org/10.1453/jeb.v6i4.1971

This paper has been cited in the "Discussion" section, in a footnote regarding the relationship between far-right voting and fear of crime. Ref: 132

Noble, J., Jardin, A. 2024. Mapping fear of crime: defining methodological orientations.Quality and Quantity, 58(2), pp. 1881–1899

This paper was already cited in the first version of the manuscript. Ref: 76

Abreu Rivera, L.F., Piatkowska, S.J. 2024. Immigrant threat, perception of unsafety, and political articulation of immigration and multiculturalism in European countries.European Journal of Criminology, 21(6), pp. 830–858

This paper has been cited in the "Discussion" section, in a footnote (the same as for Coccia, 2019) regarding the relationship between far-right voting and fear of crime. Ref: 129

Coccia M. 2017. A Theory of general causes of violent crime: Homicides, Income inequality and deficiencies of the heat hypothesis and of the model of CLASH, Aggression and Violent Behavior, vol. 37, November-December, pp. 190-200, <https://doi.org/10.1016/j.avb.2017.10.005>

This study focuses on the link between crime and income inequality. It corroborates one of our findings, which shows that the 'unworried' are overrepresented in regions with the lowest income inequality. Therefore, we have cited this article in the 'Discussion' section. Ref: 124

Uding, C.V., Porter, L.C., Dong, B., Moon, H.R. 2024. Violence, place, and health: A review of the literature, Aggression and Violent Behavior, 78, 101983

The article is quite distant from our topic. First, it does not focus on fear of crime. Second, it deals with very fine geographic scales (microplaces), whereas our study is focused on the entire country of France, with a sample that does not allow for analysis at a scale lower than that of large regions (ZEAT). Therefore, we did not cite this article.

Coccia, M., Cohn, E.G. & Kakar, S. How immigration, level of unemployment, and income inequality affect crime in Europe. Crime Law Soc Change (2024). <https://doi.org/10.1007/s10611-024-10144-y>

This study focuses on the link between crime and income inequality. It corroborates one of our findings, which shows that the 'unworried' are overrepresented in regions with the lowest income inequality. Therefore, we have cited this article in the 'Discussion' section. Ref: 125

Roman, C.G., Chen, R., Natarajan, L., ... Glanz, K., Sallis, J.F. 2024. Crime-related perceptions and walking for recreation inside and outside one's home neighborhood. Health and Place, 89, 103316

We cited this article in the "Limitations" section regarding the link between routine activities and fear of crime. Ref: 149

**Reviewer #2: Please revise the paper as per the following comments:**

1. The paper identifies two new respondent classes, but the comparison with previous versions of the EEF model is not fully detailed.

We extensively revised the "Discussion" section, where we compare the results of our model with those of previous versions. In addition to a significant reorganization aimed at enhancing the clarity and understanding of our argument, we introduced bullet points to highlight the differences between our results and those of prior studies. Finally, we included a table (Table 8) that briefly summarizes the key results of our model in comparison to those of earlier versions.

2. The results section discusses variability in sociodemographic predictors but does not provide detailed insights into the significance or effect sizes of these predictors.

To address this observation, we expanded on our hypotheses in the "Limitations" section to explain the low R² values in the regression analysis. We provide a more detailed explanation of the relationship between the observed individual effects on one hand and the overall explained variance on the other. Finally, we propose hypotheses for increasing the variance explained by sociodemographic factors in future studies.

3. While the implications for public policy are mentioned, they are not elaborated sufficiently.

The section on policy implications has been strengthened. We added several sentences emphasizing the specificities of different public policies to better demonstrate how they could address the characteristics of each class. Finally, we highlight the necessity of no longer conceiving fear of crime as a dichotomous phenomenon (‘worried’ / ‘not worried’), as this could lead to ineffective action.

4. The paper focuses on a French dataset but does not discuss how the findings might generalize to other cultural or geographical contexts.

In the conclusion, we added a section titled 'Ideas for Future Research.' Here, we discuss the possibility of generalizing the results beyond the French context. We hypothesize that these four classes are present in other geographic contexts, particularly in neighboring European countries. We base this on the results obtained in previous versions of the EEF model conducted in the United Kingdom and Germany. However, we emphasize the need to replicate these analyses in other countries, particularly in non-Western countries, to see how these relationship structures are organized in very different geographical and cultural contexts.

5. The results section discusses variability in sociodemographic predictors but does not provide detailed insights into the significance or effect sizes of these predictors.

To address this observation, we expanded on our hypotheses in the "Limitations" section to explain the low R² values in the regression analysis. We provide a more detailed explanation of the relationship between the observed individual effects on one hand and the overall explained variance on the other. Finally, we propose hypotheses for increasing the variance explained by sociodemographic factors in future studies.

6. PLOS authors have the option to publish the peer review history of their article ([what does this mean?](https://track.editorialmanager.com/CL0/https:%2F%2Fjournals.plos.org%2Fplosone%2Fs%2Feditorial-and-peer-review-process%23loc-peer-review-history/1/010f01946af6afc3-35e99ea9-96d7-4b94-a738-eb6045bd96ac-000000/hCz9zERuYQXDx7_7ieYReLEU4SUwD7JdDI-RzUyiGQ0=193)). If published, this will include your full peer review and any attached files.

If you choose “no”, your identity will remain anonymous but your review may still be made public.

**Do you want your identity to be public for this peer review?** For information about this choice, including consent withdrawal, please see our [Privacy Policy](https://track.editorialmanager.com/CL0/https:%2F%2Fwww.plos.org%2Fprivacy-policy/1/010f01946af6afc3-35e99ea9-96d7-4b94-a738-eb6045bd96ac-000000/6oG4ENc6m8zbdYug3JaI3_jRvQfBSJ7crmX_tK4Ut-U=193).

Reviewer #1: No

Reviewer #2: No
